# Analysis of spatial planning documents and strategic environmental assessment reports with regard to hazards of major industrial accidents: a case study involving six Polish cities

**DOI:** 10.1007/s11356-020-08346-7

**Published:** 2020-03-16

**Authors:** Maria Teresa Markiewicz

**Affiliations:** Faculty of Building Services, Hydro and Environmental Engineering, Warsaw University of Technology, ul. Nowowiejska 20, 00-653 Warsaw, Poland

**Keywords:** Dangerous substance, Hazardous establishment, Sensitive environment element, Risk assessment, Safety distance, Land-use planning

## Abstract

In the event of an accident in an industrial plant, the damage caused by it can be enormous. There may be environmental contamination in a large area. Injured persons may be both employees of the plant staying on its premises and local residents. The control of major accidents is realised by implementing the procedures regarding: safety analysis, emergency and operational planning, effective land-use planning (LUP), strategic environmental assessment (SEA), environmental impact assessment (EIA) and public consultation. The paper presents findings of a case study aiming to analyse selected spatial planning documents prepared at the municipality and SEA reports accompanying them, with regard to hazards of major industrial accidents in six Polish cities. In addition, changes of relevant Polish legislation taking place from 1995 till now are explained referring to European Union (EU) law. This article is the very first to present the situation concerning LUP around hazardous plants in Poland based on such extensive data. The assessment shows that the way of recording the major accident hazards is varied, but legal norms, binding at the time when the documents were drawn up, were met in each of the analysed documents. Changes of relevant Poland legislation were not the only reason for differences in the ways of recording the hazards of major industrial accidents in these documents. The case study has revealed that relevant Polish regulations can be still improved. The results of the study have useful implications for the control of major accidents, spatial policy-making and environmental management.

## Introduction

Industrial activities, carried out on a large scale, bring significant benefits to society. However, this activity also has undesirable effects on people and the environment. Contamination of the environment, to which industrial plants contribute to a large extent, leads to the violation of historically shaped ecological systems. For the population living near industrial centres, the worst felt effect of pollution emission into the environment is the deterioration of the air’s quality. In the event of an accident in an industrial plant, the damage caused by it can be enormous. There may be environmental contamination in a large area. Injured persons may be both employees of the plant staying on its premises and local residents. Such situations have occurred in the past during accidental releases of dangerous substances with toxic or flammable properties, or both. Major accidents do not occur every day, but they must be expected to occur during the production, storage, use and transport of dangerous substances. In the past, the particular concern and attention of public and state authorities, and above all the management bodies, were drawn by industrial accidents in Flixborough (the United Kingdom, 1982), Seveso (Italy, 1976), Bohpal (India, 1984), San Juanico Ixhuatepec (Mexico, 1985), Basel (Switzerland, 1986), Baia Mare (Romania, 2000), Enschende (the Netherlands, 2000) and Toulouse (France, 2001) (Tőrők et al. 2019). As a lesson learned from these accidents, special legislation acts on the international, European and national levels have been initiated and are under the process of continuous changes in order to prevent accidents and limit their consequences for man and the environment (Vierendeels et al. 2011). Since 1992 the United Nations (UN) Convention on the transboundary effects of industrial accidents, known as the Helsinki Convention (UN 1992), has been the most important document at the international level in this matter. In EU, legislation on the control of major accident hazards has got a tradition lasting nearly forty years, and it has been regulated by the so-called Seveso Directives. Since 1 June 2015, these regulations are covered by the Directive 2012/18/EU known as the Seveso III Directive (Official Journal of Laws of European Union (EOJL) 2012). In each of EU Member States (MSs), national legislation had to be harmonised with international and European regulations. The control of major accidents in industrial plants is currently realised by implementing the procedures regarding: safety analysis, emergency and operational planning, effective LUP, SEA, EIA and related to them public consultations which have been established on the basis of legal regulations. Similar regulations have been established in many non-MSs (for example Vinnem 2010; Wang et al. 2018; Anjana et al. 2018).

This paper addresses the issue of LUP around hazardous installations, which was introduced into EU legislation on 4 February 1997 by the Directive 96/82/EC (the Seveso II Directive) (EOJL 1997) and has remained in the Seveso III Directive (EOJL 2012) with minor modifications. The general idea of these regulations is to maintain the appropriate distances between the hazardous installations and the sensitive areas (such as residential areas, public spaces, main traffic roads, recreational areas or other areas of particular interest or sensitivity). Different aspects of the issue of LUP around hazardous establishments have been extensively discussed in the international scientific literature. A series of papers has reported on the transposition of EU legislation into national legislation (Mitchison 1999; Pineau 1999; Sumption 1999; Bottelberghs 2000; Demichela et al. 2014). Christou et al. (1999) and Kirchsteiger (1999) have reviewed hazard/risk assessment methods supporting the LUP decisions around the Seveso establishments adopted in MSs. Generally, three main approaches have been identified (Christou et al. 1999): a generic-safety distance approach, in which safety distances are determined depending on the type of activity and not based on the detailed analysis of the specific site; a consequence-based approach, in which consequences of credible accidents are assessed, without explicitly quantifying the likelihood of these accidents; and a risk-based approach, in which the quantitative risk assessment (QRA) is carried out. A series of papers has discussed the modification of existing LUP methodologies or development of new methodologies in particular MSs (Hirst 1998; Francis et al. 1999; Carter and Hirst 2000; Ale 2002; Salvi and Gaston 2004; Hauptmanns 2005; Cahen 2006; Cozzani et al. 2006; Tixier et al. 2006; Kontič and Kontič 2009; Sebos et al. 2010; Taveau 2010; Lenoble and Durand 2011; Delvosalle et al. 2011; Khahzad and Reniers 2015; Delvosalle et al. 2016; Török et al. 2019). Advantages and limitations of the QRA methods and its future in relation to LUP have been sketched by Pasman and Reniers (2014). Christou and Matarelli (2000) and Grønberg (2000) have described the results of the LUPACS (Land Use Planning and Chemical Sites) project aiming at the consolidation, implementation and validation of the methodology developed in LSIRIS (Laboratory of Systems and Industrial Safety) (Papazoglou et al. 1998; Papazoglou et al. 2000a; Papazoglou et al. 2000b) for LUP around major hazard facilities. Salvi and Debray (2006) have summed up general features of another methodology of risk assessment for industries developed within the ARAMIS (Accidental Risk Assessment Methodology for Industries in the framework of Seveso II directive) project, and their co-workers have characterized its details (Kirchsteiger 2002; Beerens et al. 2006; de Dianous and Fiévez 2006; Gowland 2006; Tixier et al. 2006) and its application (Kontič et al. 2006). Egidi et al. (1995) and Spandoni et al. (2000) have analysed the major accident risks connected with industrial and transportation activities in the Ravenna Area within the ARIPAR (Analisi e controllo dei Rischi Industriali e Portuali dell’Area di Ravenna – Analysis and control of the Industrial and Harbour Risk in the Ravenna Area) project. A series of papers has presented details on the technical database of accident scenarios (Christou et al. 2011; Gyenes et al. 2011; Tugnoli et al. 2013). Fabri and Wood (2019) have reported on the results of validation of the EU Commission modelling tool for consequence assessment of accident scenarios named ADAM (Accident Damage Analysis Module). A series of papers has analysed the role of GIS (Geographic Information Systems) in the LUP decision-making process (Brazier and Greenwood 1998; Contini et al. 2000; Basta et al. 2007). A review of the literature indicates that none of the publications has referred to the situation in Poland and none has commented upon the way of recording the hazards related to major industrial accident in spatial planning documents prepared in MSs.

The main goal of the paper is to describe findings of a case study aiming at the analysis of selected spatial planning documents prepared at the municipality level in Poland and related to them SEA reports with regard to hazards related to major industrial accidents. The case study involved six Polish cities whose population exceeded 450,000 inhabitants in each case. Two types of spatial planning documents were examined: a study of the conditions and directions for the municipality’s spatial development (the study) and a land-use development plan (the plan). In order to conduct the case study, the changes of Polish legislation on LUP around hazardous establishments involving dangerous substances (related directly to the analysed matters), taking place from 1 January 1995 till now, have been reviewed referring to the EU law (Evolution of Polish legislation on LUP around hazardous plants in the period from 1995 till now referring to the EU legislation). Among other things, the proposal of Polish legal regulations on the hazard/risk assessment method supporting the LUP decisions around hazardous plants is explained. Poland belongs to MSs which are still looking for the optimal solution in this matter. Subsequently the findings of the case study are discussed (Case study), and finally, this author’s reflection on a possible further development of Polish legislation on LUP around hazardous plants is presented (Conclusion and a discussion of the results). This article is the very first to present, basing on so extensive data, how the hazards related to major industrial accidents are recorded in the spatial planning documents and SEA documents prepared at municipality level in Poland. The applied methodology was tested and approved by carrying out a preliminary analysis of spatial planning and SEA documents for the city of Poznań (Markiewicz 2015).

## Evolution of Polish legislation on LUP around hazardous plants in the period from 1995 till now referring to the EU legislation

### EU background

The control of major accident hazards has been introduced to EU legislation by Directive 82/501/EEC (the Seveso I Directive) (EOJL 1982) on 24 June 1982. A deadline for implementation of this regulation to MSs legislation was 8 January 1984. As far as the issue of LUP in the vicinity of hazardous plants involving dangerous substances is concerned, it has been introduced to EU legislation by the Seveso II Directive (EOJL 1997) that entered into force on 4 February 1997 and had to be implemented into MSs legislation within two years from this date. Art. 12 of this Directive required MSs to take into account in their land-use policy (and/or other relevant policies) the objectives of preventing major accidents and mitigating the consequences of such accidents through controls on the siting of new establishments, modifications to existing establishments and new developments. It also required MSs to consider, within their land-use policy, the need to establish and maintain appropriate separation distances between the establishments covered by this Directive and the sensitive areas, and for additional measures in existing establishments so as not to increase the risk to people. In addition, Art. 12 required MSs and all competent authorities and planning authorities to set up appropriate consultation procedures to facilitate the implementation of these land-use policies. In order to help the competent authorities comply with Art. 12 of the Seveso II Directive (EOJL 1997), the document called the “Guidance on LUP as required by Council Directive 96/82/EC” (Christou and Porter 1999) was elaborated in 1999. It referred to the use of existing technical approaches and to procedural issues (Christou and Matarelli 2000). In 2003, the provisions on LUP were developed by Directive 2003/105/EC (the Amending Seveso II Directive) (EOJL 2003) which entered into force on the day of its publication, and the MSs had to bring into force the laws, regulations and administrative provisions necessary to comply with this Directive before 1 July 2005. The amendment in Art. 12 further emphasised the need to develop common guidelines by calling for the development of a common database, to be used in order to assess the compatibility between the Seveso establishments and their surroundings. As a result, MSs were provided with three other documents: the new “Guidelines” (Christou et al. 2008) defining principles for dealing with the requirements of Art. 12 in operational terms, the “Roadmaps” document (Basta et al. 2008) with supplementary information material describing in detail “good LUP practices” available within selected MSs, and the technical database of common scenarios, failure frequencies and data (Gyenes et al. 2017) to be used as a common reference for assessing the hazard/risk. The Seveso III Directive (EOJL 2012) which entered into force on 13 August 2012 and repealed the Seveso II Directive on 1 June 2015 generally contains the same LUP philosophy as the amended Seveso II Directive (EOJL 2003). It has sustained all provisions on the control of major accident hazards involving dangerous substances related to LUP which were included in the amended Seveso II Directive (EOJL 2003) except for the one concerning the elaboration of the guidelines as they had already been elaborated and adopted by the Commission. However, the content of Art. 12 of the amended Seveso II Directive (EOJL 2003) has been moved to Art. 13 of the Seveso III Directive (EOJL 2012). The minor modifications of the said LUP provisions can be characterised as follows. It has been clarified that the requirements apply to all establishments covered by the Directive and are aimed to protect both man and the environment, including areas of particular natural sensitivity. Reference has been made to procedures under Directive 2011/92/UE (EOJL 2011) (the EIA Directive) and Directive 2001/42/EU (EOJL 2001) (the SEA Directive) and other similar EU legislation; it has been stated that MSs “may provide for coordinated or joint procedures in order to fulfil the requirements of Art. 13 and the requirements of that legislation, inter alia, to avoid duplication of assessment or consultation”. The requirements have been added that MSs have to ensure that operators of lower tier establishments (LTEs) provide, at the request of the competent authority, sufficient information on the likelihood of a major accident, its potential consequences and its extent necessary for LUP purposes. It is important to notice that in the case of upper tier establishments (UTEs) such information has to be included, among other information, in the safety report obligatorily produced for this type of establishments. This requirement has been introduced in Art. 10 of the Seveso III Directive (EOJL 2012).

The risk as central for LUP purposes is also considered in the SEA Directive (EOJL 2001) which entered into force on 21 July 2001. It has been required to implement this directive in MSs before 21 July 2004. Its aim is to ensure that the environmental consequences of certain plans and programmes are identified and assessed during their preparation and before their adoption. This requirement covers, among other things, plans and programmes which are prepared for town and country planning as well as land-use (Art. 2(2a)). The information which has to be provided for the purpose of SEA process includes, among other things, “likely significant effects on the environment, including on issues such as biodiversity, population, human health, fauna, flora, soil, water, air, climatic factors, material assets, cultural heritage including architectural and archaeological heritage, landscape and the interrelationship between the above factors” (point “f” in Annex 1). Although the issue of major accident hazards is not explicitly referred to it in the SEA Directive (EOJL 2001), it can be considered that this provision covers this issue.

### Situation in Poland

In Poland, legislation on LUP around hazardous plants involving dangerous substances has got a tradition lasting over thirty years and has been regulated mainly by legal acts regarding environmental protection, spatial planning and environmental impact assessment (Table 1). The regulations on the control of major accident hazards have been included in the legal acts on environmental protection. In the 1990s, they differed from EU regulations, but in 2001 (i.e. three years before Poland’s accession to the EU, which took place on 1 May 2004) they were fully harmonised with the Seveso II Directive (EOJL 1997) (Markowski 2005; Pszczel 2009). In the subsequent years, the changes introduced to Polish legislation concerning the issue of LUP around the hazardous plants were the result of development of EU regulations (in particular the amending Directive (EOJL 2003) and the Seveso III Directive (EOJL 2012)). In this section, legislation concerning the issue of LUP around hazardous plants binding in Poland from the beginning of 1995 till now (related directly to the matters analysed in the case study) is reviewed referring to EU legislation presented in EU background. The chosen period overlaps with the period during which the analysed spatial documents were drawn up (Tables 3 and 4 in Case study).

**Table 1:** Polish legal acts covering environmental protection, spatial planning and environmental impact assessment since 1995 till now

Binding period	Name of the legal act/reference	Notation in PJL
1 October 1980–1 October 2001	Act of 31 January 1980 on Protection and Management of the Environment (the PME Act) (PJL 1980, 1994b)	(PJL from 1980, no 3, item 6, with amendments; PJL from 1994, no 49, item 196)
1 January 1995–11 July 2003	Act of 7 July 1994 on Land-use Management (the LUM Act) (PJL 1994a)	(PJL from 1994, no 89, item 415, with amendments)
1 January 2000–1 October 2001	Act of 9 November 2000 on Access to Environmental Information and Its Protection and on Environmental Impact Assessment (the AEIA Act) (PJL 2000)	(PJL from 2000, no 109, item 1157, with amendments)
1 October 2001–till now	Act of 27 April 2001 on Environmental Protection Law (the EPL Act) (PJL 2001a, 2019)	(PJL from 2001, no 62, item 627, with amendments; PJL from 2019, item 1396, consolidated version)
11 July 2003–till now	Act of 27 March 2003 on Spatial Planning and Land-use Management (the SPLUM Act) (PJL 2003a, 2018a)	(PJL from 2003, no 80, item 717, with amendments; PJL from 2018, item 1945, consolidated version)
15 November 2008–till now	Act of 3 October of 2008 on Making Available Information about Environment its Protection, the Public’s Participation in Environmental Protection, as well as on Environmental Impact Assessment (the MEIA Act) (PJL 2008a, 2018b)	(PJL from 2008, no 199, item 1227, with amendments; PJL from 2018, item 1396, consolidated version)

The implementation of the Seveso II Directive (EOJL 1997) into Polish regulations took place through the publication in 2001 of a new act on environmental protection – the EPL Act (PJL 2001a). In the context of the analysis of the spatial planning and SEA documents described in Case study, the following changes introduced by this act should be noted:

1. Before 2001, the term “unusual environmental hazard” (a hazard created by an unexpected occurrence different from a natural disaster which may lead to a serious danger to human life and the environment) defined in the PME Act (PJL 1994b) had been used. Since 2001, the terms “major accident” (an occurrence, in particular an emission, fire or explosion, created during operational industrial activities, storage or transport, and leading to a serious danger to human life and health or the environment, immediate or delayed, and involving one or more dangerous substances) and “major industrial accident” (a major accident at an establishment) have been used. The term “major accident” covers a broader spectrum of events than its definition given in the successive Seveso Directives.
2. Before 2001, the term “stationary installations that may create unusual environmental hazards” (referred to as installations located in buildings or at fixed places, in which production, handling, processing, storage, transport and use of dangerous substances take place in such form and quantities that it may lead to major accidents or disasters involving these substances) defined in the PME Act (PJL 1994b) had been used. Since 2001, the terms “lower-tier establishment” (LTE) and “upper-tier establishment” (UTE) have been used.
3. From 1998 to 2001, based on the amendment to the PME Act (PJL 1994b) (introduced by Act of 29 August 1997 amending the Act on Protection and Management of Environment and Other Acts (PJL from 1997, no 133 item 885) (PJL 1997)) for a stationary installation that may create an unusual environmental hazard, there was an obligation of drawing up a safety report (Art. 105b.4) and emergency plans describing the activities carried out within and outside the plant area (Art. 105a.1 and Art. 105b.1). Since 2001, based on the EPL Act (PJL 2001a) for the LTE, it has been required to prepare a major accident prevention programme (Art. 25.1) (in the successive Seveso Directives this document has been called a major accident prevention policy) and a safety management system (Art. 252.1). For the UTE, in addition to the mentioned documents, it has been required to draw up a safety report (Art. 253.1) and internal and external emergency plans (Art. 260.1).
4. For the entire period considered, the construction of plants which have been dangerous to human life or health has been banned within administrative boundaries of the cities and near the compact development of villages. Before 2001, this was regulated by Art. 73.1(2) of the PME Act (PJL 1980, 1994b). Since 2001, it has been regulated by Art. 73.3 of the EPL Act (PJL 2001a). In addition according to Art. 73.3 of this act, the extension of these plants has been acceptable under the condition of causing the reduction of danger to human health, in particular the reduction of creating major accidents.
5. Before 2001, based on Art. 73.1(1) of the PME Act (PJL 1980, 1994b;) the construction of buildings that would be a danger to the environment, and introducing modifications to the existing buildings, had been forbidden in the areas requiring special protection. In addition, based on Art. 73.3 of the PME Act (PJL 1980, 1994b), it had been required to take into account in the plans and decisions on land development conditions certain restrictions concerning the location of production plants which would be a danger to human health. Since 2001 based on Art. 73.4 of the EPL (PJL 2001a) in the case of the location of a new plant posing major accident hazards, it has been required to keep a safety distance from other plants creating major accident hazards and vulnerable areas.

In the period from 2003 till 2015, when the amended Seveso II Directive (EOJL 2003) was binding, the following changes important for the analysis in Case study have been introduced to Polish legislation:

1. Since 2003, as a result of amendment to the EPL Act (PJL 2001a) (introduced by Act of 3 of October 2003 amending the Act on Environmental Protection Law and other Acts (PJL from 2003, no 190, item 1865) (PJL 2003b)), the construction of new plants creating major accident hazards and introducing modifications to existing plants of this type has been permitted in the areas delimited as the terrains of production facilities, warehouses and storage facilities in the plans (if the plans do not involve restrictions concerning establishments posing a threat to human health and life) (Art. 73.3a).
2. For the entire period considered, the requirements of health care and the safety of people and property had to be taken into account in LUP. From 1995 till 2003, it was regulated by the LUM Act (PJL 1994a) in the form of a general postulate (Art. 1.2(3)). Since 2003, this issue has been regulated by the SPLUM Act (PJL 2003a) in the form of a general requirement (Art. 1.2(5)) and in addition in the form of a detailed requirement in relation to the study (Art. 10.1(6)) and the plan (Art. 15.1(1)).
3. Since 2006, as a result of the amendment to the EPL Act (PJL 2001a) (introduced by Act of 24 February 2006 amending the Act on Environmental Protection Law and other Acts (PJL from 2006, no 50, item 360) (PJL 2006a)), in the case of the location of new vulnerable investments in the vicinity of existing plants that create major accident hazards, it has been necessary to maintain a safety distance (Art. 73.5). In the case of an existing plant located without keeping the safety distance, the Provincial Inspector for Environmental Protection, after obtaining the opinion of the competent authority of the State Fire Service (SFS) (the Provincial Commandant of the SFS in case of the UTE and the District Commandant of the SFS in case of the LTE), has been entitled to request implementing additional technical measures (Art. 73.6), in order to increase people’s safety.
4. Since 2007, it has been recommended to determine the safety distance for LUP purposes in Poland using a generic-distances approach, described in the Polish guidelines (Małaczyński et al. 2007, 2010). In complex cases, it has been suggested that the safety distance should be determined on a case-by-case basis, but no information has been provided on the method of conducting the analysis. In the case of areas designed for industry in which Seveso establishments can be located, an extended description has been proposed, clearly informing about such land-use of this area. It is important to notice that it has not been mandatory to fulfil the guidelines.
5. Since 2010, as a result of amendment to the SPLUM Act (PJL 2003a) (introduced by Act of 25 June 2010 amending the Act on Spatial Planning and Land-use Management, the State Sanitary Inspection Act and the Monument Protection Act (PJL from 2010, no 130, item 871) (PJL 2010)), the competent authority of the SFS and the Provincial Inspector of Environment Protection have been obliged to agree on a draft of the study with regard to siting a plant that may create major accident hazards as well as the location of the residential areas and public spaces in the vicinity of existing plants that may create major accident hazards, and to express the opinion on a draft of the plan in these matters (Art. 11.6 and Art. 17.6).

The Seveso III Directive (EOJL 2012), which replaced the amended Seveso II Directive, was implemented in Poland in 2015 by the Act of 23 July 2015 amending the Act on the Environmental Protection Law and other Acts (PJL from 2015, item 1434) (PJL 2015). In the context of the analysis from Case study, the following changes introduced to the EPL Act (PJL 2001a) in 2015 are important:

1. The operator of the LTE is put under an obligation to prepare and provide information on the likelihood of a major accident, its consequences and its range for LUP purposes (Art. 73.7).
2. The competent authority of the State Fire Service (SFS) is obliged to agree on the decision on land development conditions with regard to siting a plant that may create major accident hazards as well as the location of the residential areas and public spaces in the vicinity of existing plants that may create major accident hazards (Art. 53.4(12) and Art. 64.1).
3. The Provincial Commandant of the SFS has to identify a group of plants that, due to their location, can create domino effects (Art. 264d).
4. Operators of neighbouring plants are required to exchange information on factors that may increase the likelihood of a major accident, aggravate its consequences or create domino effects (Art. 259).
5. A delegation concerning the preparation of the ordinance on safety distance determination has been introduced to the EPL Act (PJL 2001a) (Art.73a). The legislative work on the draft of this ordinance has been carried out since 2015. In the third version of the draft of ordinance from 26 March 2019 on safety distance determination (Governmental Legislative Centre (GLC) 2019), a consequence-based approach is used. It is proposed to consider nine reference scenarios of possible accidents, one set of meteorological conditions (meteorological stability D, wind velocity 5 m/s) and a local aerodynamical roughness coefficient. No reference mathematical model for the calculation of hazardous zone range is given. It is proposed to assess consequences of accidents by calculating the distances in which the impact on human health and/or physical effects reaches the set threshold values. In this way, five zones can be defined, according to which LUP restrictions are specified.

In addition, in the context of the analysis of the spatial planning and SEA documents described in Case study, it is important to notice the following:

1. In the considered period (since 1995 till now), the executive acts (ordinances) supplementing legal acts have been modified, or new versions of the ordinances have been issued. Notification on major accidents to the Chief Inspector of Environmental Protection has been regulated by succeeding versions of the ordinance (PJL 2003c, 2016c). Categorisation of establishments has been regulated by successive versions of the ordinance (PJL 2002a, 2006b, 2013, 2016a). Contents of the emergency plans and safety reports have been regulated by succeeding versions of ordinances PJL 2001b, 2001c, 2003e, 2008b, 2016d and PJL 2001d, 2003d, 2005, 2016b. In the ordinance on the content of the safety report (PJL 2016d) (issued on the basis of the amendment to the EPL Act (2001a) introduced by the Act (PJL 2015)), there is an obligation to include information for LUP purposes in the safety report (Section 3.8(11)).
2. For the entire period considered, there has been an obligation to prepare a SEA report for the plan. From 1995 till 2002, the ordinance (PJL 1995) was binding in which the issue of major accident hazards has been explicitly included. From 2000 to 2001, the scope of the SEA report was specified by Art. 20(2) of the AEIA Act (PJL 2000), and in addition, the scope of the SEA report for the plan was regulated in the ordinance (PJL 1995) issued on the basis of the LUM Act (PJL 1994a). Similar regulations were binding from 2001 to 2008, i.e. the requirements to be met by the SEA report were specified by Art. 41(2) of the EPL Act (PJL 2001a), and in addition, the scope of the SEA report for the plan was regulated by the ordinance (PJL 2002b) which was issued on the basis of Art. 41(3) of this Act. Since 2008, the scope of the SEA report for LUP documents has been regulated in Art. 51.2 of the MEIA Act (PJL 2008a). The issue of major accident hazards is not explicitly included in it. However, it can be considered that this issue is implicitly referred to in Art.51.2(2e) of this act. It requires that the SEA report identifies, analyses and assesses the potential impacts of the provisions of spatial planning document draft on the purposes and objects of protection of the Natura 2000 area and integrity of this area, as well as on the environment, and in particular on: biodiversity, population, fauna, flora, water, air, the surface of the earth, landscape, climate, natural resources, monuments, material goods, taking into account the interrelationship between these elements. In Art. 52.3 of the MEIA Act (PJL 2008a), a delegation was attached to issue an ordinance on additional requirements to be met by the SEA report for a draft of the plan, but this executive regulation has not yet been issued The obligation to prepare the SEA report for the study was introduced in 2008 by Art. 46.1 of the MEIA Act (PJL 2008a).
3. Since 2015, as a result of amendment to the SPLUM Act (PJL 2003a) (introduced by Act of 23 July 2015 amending the Act on the Environmental Protection Law and other Acts (PJL from 2015, item 1434) (PJL 2015), the competent authority of the State Fire Service (SFS) has been obliged to agree on the decision on land development conditions with regard to siting of a plant that may create major accident hazards as well as the location of the residential areas and public spaces in the vicinity of existing plants that may create major accident hazards (Art. 53.4(12) and Art. 64.1).

It should also be noted that in Poland, the main idea is that the plan provides only general terms and conditions for the location of specific investments (also the Seveso establishments). Implementation of the plan is a necessary condition, but not sufficient to ensure protection and proper use of the environment. Achieving this goal will be effective only with full coordination of efforts of all participants in subsequent decision-making processes related to the construction process. Polish legislation related to the construction process, in particular the role of EIA of a specific investment, does not fall within the scope of this paper. More information in English on this subject can be found in Woloszyn (2004) and Borowski and Walas (2016).

## Case study

### General information

The case study involved Warszawa (Warsaw), Kraków (Cracow), Poznań, Wrocław, Gdańsk and Łódź. The basic research question was how were hazards of major industrial accidents recorded in the provisions of spatial planning documents prepared at the municipality level and the accompanying SEA reports for the said documents. Two types of spatial planning documents were examined: the studies and the plans. The study determines spatial policy in a municipality. It is an obligatory document but not legally binding, prepared for the whole municipality area. The plan constitutes an essential tool to implement municipality’s spatial policy formulated in the study. The local council adopts the plan in the form of a local by-law. It might be prepared for the whole municipality area or its part. Areas for which it is obligatory to prepare the plans are regulated by law. There is no obligation to draw up the plan if the Seveso establishments are present in the area or their siting is planned. In the absence of the plan, local authorities can manage spatial development through ordering a decision on land development conditions that is binding upon authorities responsible for issuing a building permit. Currently, binding LUP documents in Poland were drawn up either based on the LUM Act (PJL 1994a) which was in force from 1 January 1995 to 11 July 2003 or the SPLUM Act (PJL 2003a) which after replacing the LUM Act (PJL 1994a) is still binding.

The implementation of the case study included the following stages:

1. defining the Seveso establishments locations in cities
2. assigning the plans to the Seveso establishments, taking into account their locations and areas covered by the plans;
3. analysis of the available documents with regard to hazards related to major industrial accidents

The spatial planning documents in selected cities were collected on the websites of the City Hall Planning Offices and/or the City Architectural Studios. The City Hall Planning Offices and City Architectural Studios, if digital versions of the SEA reports were available, sent these documents on the written request to the author. The case study started at the beginning of 2014 and lasted until the end of 2016. All findings were checked at the end of this period, taking into account the list of Seveso establishments from the beginning of 2016 which was sent by the Chief Inspectorate of Environmental Protection at the written request of the author. In this publication, all findings are presented in a very concise form. It should be added that after completing the case study, the drafts of new versions of the studies were available for Łódź, Wrocław and Gdańsk. They were examined at the beginning of 2018 to enlarge the analyses.

Statistical information on the Seveso establishments and the plans in each of the considered cities are presented in Table 2. The dates of passing the resolutions concerning the studies and the availability of the SEA reports for the studies are given in Table 3. The plans assigned to the Seveso establishments are presented in Table 4. Taking into account that at the beginning of 2016 in the considered cities, there were thirty-one Seveso establishments, fourteen plans in force (of which one plan covered two Seveso establishments) and one plan was under development; it can be calculated that for fifteen Seveso establishments (approximately 50%), no plans were adopted and no plans were being drawn up.

**Table 2:** Statistical information on the Seveso establishments, the plans in force and the plans under development in each of the considered cities

City	Warszawa	Kraków	Poznań	Wrocław	Gdańsk	Łódź
Areas covered by the plans in force/under development [%]^1^	36/35	49/29	41/27	57/5	64/5	22/16
Numbers of plans in force/under development covering the Seveso establishment location area [−]	2/0	0/1	3/0	2/0	6/0	1/0
Numbers of UTEs and LTEs [−]^2^	0/7	1/2	0/5	2/3	3/4	1/3

^1^Information obtained on a written request from the City- Hall Planning Offices

^2^Information obtained on a written request from the Chief Inspectorate of Environmental Protection

**Table 3:** Information on the studies and the SEA reports for the studies in the chosen cities

City	Dates of passing the study and revisions to the study	Reference	Availability of the SEA report
Warszawa	10.10.2006; 6.02.2009; 28.04.2009; 7.10.2010; 11.07.2013;16.10.2014	Warszawa City Council (WCC 2014)^3^	–
Kraków	16.04.2003; 3.03.2010; 9.07.2014	Kraków City Council (KCC 2014)	+
Poznań	23.11.1999; 8.01.2008; 23.09.2014	Poznań City Council (PCC 2014)	+
Wrocław	30.01.1998; 6.07.2006; 20.05.2010	Wrocław City Council (WrCC 2010)^4^	+
Gdańsk	20.12.2001; 20.12.2007	Gdańsk City Council (GCC 2007)^4^	-^5^
Łódź	3.04.2002; 27.10.2010	Łódź City Council (ŁCC 2010)^4^	–

^3^In 2002, the administration area of Warszawa was changed, and a new study covering the new area was adopted in 2006. In the period from 9 July 2001 till 10 October 2006 the spatial planning document entitled the “Spatial development plan for the capital city of Warszawa with the agreements binding Warszawa municipalities when drawing up the land-use development plans” acted as the study (WCC 2001)

^4^In the end of 2017, the drafts of new versions of the studies for Wrocław (WrCC 2017), Gdańsk (GCC 2017) and Łódź (ŁCC 2017) were available

^5^An obligation to prepare a SEA report for the study was introduced in 2008

**Table 4:** Information on the plans covering the areas of the Seveso establishments’ locations and the SEA reports in the chosen cities

The plan			Dates of passing resolutions on drawing up and accepting the plan	Seveso establishment	
Name	Reference	SEA report^6^		Type	Qualification
Warszawa					
“Targówek Przemysłowy”	WCC 2000	–	27.04.1995–27.04.2000	LTE	2007
“Mory i Karolin, Glinianki Sznajdra”	WCC 2002	–	16.05.1996–14.03.2002	LTE	2014
Kraków					
“Balice II”	KCC 2016		22.10.2016 – under development	LTE	2002
Poznań					
“Malta”	PCC 2002	+	10.05.1994–23.04.2002	LTE	2002
“Janikowo 1”	PCC 2008	+	6.03.2007–4.11.2008	LTE	2007
“Naramowice – ul. Karpia”	PCC 2009	+	2.03.2004–7.04.2009	LTE	2009
Wrocław					
“Kwidzyńska”	WrCC 2012	+	5.10.2006–28.12.2012	LTE LTE	2008 2002
“Robotnicza i Góralska”	WrCC 2013	+	3.07.2012–28.11.2013	LTE	2013
Gdańsk					
“Młyńska Letnica”	GCC 2002a	–	17.06.1998–21.02.2002	LTE	2002
“Przeróbka”	GCC 2002b	+	15.06.1999–26.09.2002	LTE	2011
“Płonia”	GCC 2006	–	24.06.2004–31.08.2006	UTE	2002
“Port Północny” part I	GCC 2009	+	24.11.2005–27.08.2009	UTE	2002
“Krakowiec – Górki Zachodnie, Kępna i Stogi”	GCC 2011	+	30.09.2010–22.12.2011	UTE	2002
“Stogi Portowe” close to “Twierdza Wisłoujście”	GCC 2014	+	25.06.2013–26.06.2014	LTE	2006
Łódź					
“Sokołów”, south part of the housing estate	ŁCC 2013	+	27.06.2007–30.10.2013	LTE	2002

^6^(+)/(−) The SEA document was/was not available to this author

### Analysis of the selected spatial planning documents prepared at the municipality level and related to them SEA reports

The analysis used a descriptive method. In particular, the following issues were examined: terminology used, reporting the existing Seveso establishments, type of land-use within the Seveso establishment, restrictions and/or approvals regarding land-use, reference to legal regulations concerning the location of hazardous plants involving dangerous substances, in particular Art. 73 of the EPL Act (PJL 2001a, 2019), determination of the safety distance, applying the method of safety distance determination and using an extended description clearly indicating the possibility of location of the Seveso establishment from the Polish guidelines (Małaczyński et al. 2007, 2010).

Considering that Polish legislation on LUP around the Seveso establishments was subject to changes in the period in which the analysed documents were being drawn up, the opportunity to investigate how these changes affected the provisions contained in the spatial planning and SEA documents was taken. It was useful to distinguish certain characteristic points on the time scale. Based on Table 1 and the discussion presented in section 2.2, four characteristic points were chosen: 1 October 2001, 15 November 2008, 21 October 2010 and 1 July 2015. The second characteristic point refers to the SEA reports, while the remaining ones to the spatial planning documents.

The analysis of the content of the studies and SEA documents for the studies in the cities considered with regard to recording hazards related to major industrial accidents leads to the following observations:

1. All analysed studies (or revisions of the studies) were prepared based on the SPLUM Act (PJL 2003a).
2. The issue of hazards of major industrial accidents was present in all analysed studies. The terminology was the same as of today, with some exceptions. In the study for Łódź (ŁCC 2010), the term of “unusual environmental hazard” was used instead of “major accident”. In none of the studies, the term “safety distance” was used.
3. Information on numbers of the UTEs and/or LTEs in the city and their locations was provided in all analysed studies. The lists of the Seveso establishments in the cities given in the studies were not necessarily identical to the list from the beginning of 2016. In addition, in the study for Poznań (PCC 2014), the range of the hazardous zones was given. It was written that “the maximum radius of contamination [around hazardous plants] varied from 0.8 to 3.5 km”. The study for Warszawa (WCC 2014) contained a map showing the locations of the Seveso establishments in the city.
4. Almost all the Seveso establishments listed in Table 4 (except the “Wyborowa S.A.” plant located in the area covered by the plan (PCC 2002) in Poznań) for which the plans were available were located in areas designed in the studies as: “industrial areas”, “areas of technical and production activities”, “areas of production activities and services”, “areas of production facilities, warehouses and storage facilities” or “areas of the economic activity” (this last group of land-use categories included production activities among nearly twenty of categories). The “Wyborowa S.A.” plant in Poznań was located in an area subject to functional and spatial transformations. On the map showing the conditions of spatial development, this area was designed as the “area of production facilities, warehouses and storage facilities”, but at the map showing the directions of spatial development, it was designed as the “sport and recreation activities area” and the “service area”.
5. In all the analysed studies, there were information about dangerous substances transported in big amounts through the city, the road and rail transport routes and parking lots. The residential areas of the greatest danger due to their location in the vicinity of the Seveso establishments or transportation routes of dangerous materials were listed.
6. In all the analysed studies, the guidance (planning solutions) was defined, allowing to reduce threats to life and health of the people and the environment resulting from industrial accidents and failures of vehicles transporting dangerous materials.
7. The SEA reports were prepared for the studies in Kraków (KCC 2014), Łódź (ŁCC 2010), Poznań (PCC 2014), Warszawa (WCC 2014) and Wrocław (WrCC 2010). The lack of the SEA report for the study for Gdańsk (GCC 2007) was explained by the fact that the study had been adopted before an obligation of drawing up such as report for the study was introduced, i.e. before 2008. The analysed SEA reports for the studies were prepared based on the MEIA Act (PJL 2008a).
8. Out of the three SEA documents of the studies available to the author (i.e. the documents for Kraków (KCC 2014), Poznań (PCC 2014) and Wrocław (WrCC 2010)), the issue of the risk of major accidents was given the most attention in the SEA report for the study for Kraków (KCC 2014).
9. In the draft of the latest version of the study for Gdańsk (GCC 2017), which had been submitted to public inspection at the end of 2017, the scope of information on the issue of major accident hazards was updated and extended (in particular the relevant regulations were clarified). In the drafts of the latest versions of studies for Łódź (ŁCC 2017) and Wrocław (WrCC 2017), information on that subject was updated. From among the three SEA reports for the drafts of the said studies, the risk of major accidents was discussed in detail in the SEA report for the draft of the study for Gdańsk (GCC 2017).
10. Neither the method of determination of the safety distance nor the extended description indicating the location of the Seveso establishments from the Polish guidelines (Małaczyński et al. 2007, 2010) were used in the analysed documents (the studies, drafts of the studies or SEA reports).

Analysis of the contents of the plans covering the areas of the Seveso establishment locations and the SEA reports for these plans with regard to recording hazards of major industrial accidents leads to the following observations:

1. Out of the fourteen analysed plans in force, the five plans were prepared based on the LUM Act (PJL 1994a). This concerned the documents (GCC 2002a, 2002b) in Gdańsk, (PCC 2002) in Poznań and (WCC 2000, 2002) in Warszawa. The remaining of the plans, i.e. (GCC 2006, 2009, 2011, 2014;) in Gdańsk, (ŁCC 2013) in Łódź, (PCC 2008; PCC 2009) in Poznań, (WrCC 2012, 2013) in Wrocław were prepared based on the SPLUM Act (PJL 2003a). The resolution on drawing up the plan (KCC 2016) in Kraków was also passed based on the SPLUM Act (PJL 2003a).
2. In the nine cases, the plant had been placed on the Seveso establishments list before the plan was accepted. This concerned the documents (GCC 2006, 2009, 2011, 2014) in Gdańsk, (ŁCC 2013) in Łódź, (PCC 2002, 2008) in Poznań and (WrCC 2012) in Wrocław. But information that the Seveso establishment was located in the area covered by the plan was given only in resolutions of two of these plans, i.e. (GCC 2011, 2014) in Gdańsk. In resolutions of three other plans in this set, information appeared that a particular plant was located in the area covered by the plan but without specification that it had been classified as the Seveso establishment. This concerned documents (GCC 2009) in Gdańsk, (PCC 2002) in Poznań and (WrCC 2012) in Wrocław.
3. In all the analysed plans in force, the land-use of terrain in the areas of the Seveso establishment locations was coordinated with provisions of the studies or other spatial planning documents acting as a study which were binding at the time of passing the plans (in Warszawa in the period from 28 September 1993 to 31 December 2003, the land use development plans had to be coordinated with the “general spatial development plan for the capital city of Warszawa” (WCC 1992) and in addition in the period from 9 July 2001 till 10 October 2006 with the document entitled the “spatial development plan for the capital city of Warszawa with agreements binding Warszawa municipalities when drawing up the land-use development plans” (WCC 2001) which acted as a study).
4. The information about the coordination of the plan with the study was explicitly stated in ten plans. There were five exceptions ((GCC 2002a, 2002b) in Gdańsk, (PCC 2002) in Poznań, (WCC 2000, 2002) in Warszawa).
5. In Gdańsk in four plans (GCC 2006, 2009, 2011, 2014), the Seveso establishments were located in the areas designed as “areas of production activities and services” (intended for all economic activities in the field of production facilities, warehouses and storage facilities as well as services including sea and river ports) and in two other plans (GCC 2002a, 2002b) in the areas designed as “industrial areas*”* (intended for large-scale production and production with a high degree of nuisance). At the same time in some plans, a ban was introduced on the location of hospitals and nursing homes, buildings for full-time or part-time stay of children (GCC 2006, 2009, 2011, 2014), commercial premises with shopping areas of over 2000 m^2^ (GCC 2002b, 2011), collective residential buildings (GCC 2006, 2009, 2011) and apartments integrally connected with the economic activity (GCC 2011). A requirement to limit the potential noxiousness of the facility to the terrain belonging to the plant was introduced in the plan (GCC 2002a).
6. In Lódź, the area of the Seveso plant location was designed as “industrial area”. Technical infrastructure and service buildings with the proviso that they do not exceed 50% of surface area, greenery and communication were introduced as a complementary land-use of terrain. At the same time, a ban was introduced on locations of commercial premises with shopping areas of over 2000 m^2^.
7. In Poznań in two plans (PCC 2008, 2009), the Seveso establishments were located in the areas designed as “areas of technical and production activities”. Communication and technical infrastructure were allowed as a complementary land-use of terrain. In the plan (PCC 2009), an obligation to isolate dangerous facilities with greenery and a ban to construct buildings that require acoustic comfort were introduced. In the resolution of the plan (PCC 2002), it was stated that, fifteen years after approval of the plan, the “Wyborowa S.A.” plant was to be transformed into the area of recreation and sports services.
8. In Warszawa, the areas of the Seveso establishments were designed in the plan (WCC 2000) as “areas of production facilities, warehouses and storage facilities” and in the other plan (WCC 2002) as “buildings and heating installations” (i.e. the existing “Wola” heating plant). Communication, technical infrastructure and greenery were allowed as complementary land-use of terrain in the plan (WCC 2000). In addition, a ban on location of residential buildings and vulnerable functions (education and health) and on cultivation of vegetables, cereals and fruits was introduced. Also, an obligation to isolate dangerous facilities with greenery was specified. In both plans, a requirement to limit the potential noxiousness of the facility to the land belonging to the plant was included.
9. In Wrocław in two plans (WrCC 2012; 2013), the Seveso establishments were located in the areas designed as “areas of the economic activity”. This group of land-use categories included many different land-use categories such as production activities, warehouses and wholesale trade, offices, communication, technical infrastructures, vehicle service, vehicle repair). As a complementary land-use of terrain, greenery and construction equipments accompanying the land-use categories authorised in this area were allowed.
10. In almost all plans (except the plans (GCC 2011) in Gdańsk and (PCC 2002) in Poznań) for areas where the Seveso plants were located, the minimum indicator of biologically active surface varied from 7 to 30%. In the plan (GCC 2011) in Gdańsk, it was equal to 0%, and in the plan (PCC 2002) in Poznań was 40%. Except the plan (GCC 2011) in Gdańsk, all other plans included an obligation to protect greenery.
11. In all analysed plans, the delineated areas in which the Seveso establishments were located covered from tens to hundreds of hectares. Functionally vulnerable areas were reasonably far removed from them. However, there were three exceptions. In the plan (PCC 2008) in Poznań, residential areas were located on the other side of the road. In the plan (PCC 2009) in Poznań, a screening green strip having a width of 10 m separated the residential area from the area where the Seveso plant was located. In the plan (WCC 2000) in Warszawa, the residential area adjoined the industrial area where the Seveso establishment was located. In the resolution of this plan, “modernisation and maintenance of residential buildings in the distinguished zone was allowed based on specific regulations”.
12. Out of the fourteen analysed plans in force, only the plan (WCC 2000) in Warszawa was adopted before 1 October 2001. From 1 October 2001 till 21 October 2010, eight plans were accepted, i.e. (GCC 2002a, 2002b, 2006, 2009) in Gdańsk, (PCC 2002, 2008, 2009) in Poznań and (WCC 2002) in Warszawa. The next five plans were accepted from 21 October 2010 to 1 July 2015. These were (GCC 2011, 2014) in Gdańsk, (ŁCC 2013) in Łódź and (WrCC 2012, 2013) in Wrocław.
13. In Gdańsk, as the only one of the considered cities, all Seveso establishments were assigned to the plans.
14. Out of the ten SEA reports for the plans available to the author, three documents were prepared before 15 October 2008. These were the SEA reports for the plans (GCC 2002b) in Gdańsk and (PCC 2002, 2008) in Poznań. They were prepared based on the LUM Act (PJL 1994a) and the ordinance (PJL 1995), or the AEIA Act (2000) and the ordinance (PJL 1995), or the EPL Act (PJL 2001a) and the ordinance (PJL 2002b), respectively. Other available SEA reports for the plans were prepared after 15 October 2008, and they were based on the MEIA Act (PJL 2008a). These were the SEA documents to the plans (GCC 2009, 2011, 2014) in Gdańsk, (PCC 2009) in Poznań, (ŁCC 2013) in Łódź and (WrCC 2012, 2013) in Wrocław.
15. Out of the ten analysed SEA reports, in six cases, the plant had been enrolled at the list of the Seveso establishments before adopting the resolution concerning the preparation of the plan to which it referred. These were the SEA reports for the plans (GCC 2009, 2011, 2014) in Gdańsk, (ŁCC 2013) in Łódź, (PCC 2008) in Poznań and (WrCC 2012) in Wrocław. But only in the SEA reports for the plans in Poznań and Gdańsk, i.e. in four cases, information was given that the plant had been enrolled at the Seveso establishments list, and the risk of major accidents was evaluated.
16. The SEA reports for the plans (PCC 2002, 2009) in Poznań and (WrCC 2013) in Wrocław gave information that the specific plant was located at the area covered by the plan, but there was no information that it was classified as the Seveso establishment.
17. The hazardous zone around the Seveso establishment was given only in the SEA document for the plan (GCC 2014) in Gdańsk. In the area covered by the plan, there was one existing Seveso plant, and the location of the new one was planned. It was stated that for a new investment “the range of impact of fire or explosion causing loss of human life was limited to about 100 m from potential sources.”
18. The SEA report for the plan (GCC 2002b) in Gdańsk gave detailed information on the plants that create unusual environmental hazards which were located at the area covered by the plan and in the close surroundings, but the specific plant located at the area of this plan currently qualified as the UTE was not mentioned. It has recently been added to the Seveso establishments list.
19. No new restrictions concerning the development of areas around the Seveso plants apart from those which were already included in the plans were introduced to the SEA documents.
20. In none of the analysed plans and the SEA documents to the plans, recommendations from the Polish guidelines (Małaczyński et al. 2007, 2010) were applied.

## Conclusions and a discussion of the results

The results of the case study confirm that the issue of noxiousness of industrial plants was present in all analysed spatial planning documents and SEA reports prepared at the municipality level in six Polish cities. The ways of recording hazards related to major accidents varied in the documents, but the legal norms binding in Poland at the time of drawing them up were met in each of the analysed documents.

The assessment of the studies and the SEA reports for the studies show that the later the spatial planning document was adopted, the more detailed information concerning the issue of major accident hazards was included in these documents. In case of the plans and SEA reports for the plans, the evaluation is not unambiguous. The ambiguity is introduced by the analysed documents from Łódź and Wrocław. Namely in two of the SEA reports for the plans adopted after 2010, no information was provided regarding the issue of major accident hazards although the plants located in these areas had been included in the Seveso establishments list prior to the adoption of the plans. Most likely, this resulted from a gap in legal regulations in Poland existing since in 2008, i.e. lack of the ordinance on the scope of the SEA report for the plan in which the problem of risk of a major industrial accident would have been clearly raised.

The obtained results suggest that the changes of Polish legislation on the control of major accident hazards that occurred in the period in which the analysed documents were drawn up, with a tendency to strengthen up these regulations, were not the only reason for differences in presenting the major accident hazards in these documents. Two other reasons for these differences seem equally important. The first one is directly related to the moment of classifying a plant as the Seveso establishment in comparison with the moment of passing the resolution regarding the adoption of the plan. The second one is related to the growing attention being paid not only by the spatial planners and SEA experts but also by representatives of the government administration and industry, researchers and the public to safety and risk assessment in relation to industrial activity.

The method of safety distance determination from the Polish guidelines (Małaczyński et al. 2007, 2010) was not effectively used in the process of drawing up the LUP and SEA documents in Poland. According to spatial planners, the reason for this was the lack of legal validity of the guidelines. The proposed ordinance (GLC 2019) on safety distance determination is a legal basis for unifying the principles of considering major accident hazards in LUP. The consequence-approach, which can be placed, regarding the complexity of analysis, between the generic-distance approach and the risk-based approach (Christou et al. 1999), is a reasonable option in Polish conditions. It would allow for the more detailed analysis than the generic distance approach (which has been adopted for example in Germany and Sweden (Christou and Matarelli 2000)) and still is not as complex as the QRA approach (which has got a long tradition in the UK (Francis et al. 1999; Carter and Hirst 2000) and Netherlands (Bottelberghs 2000; Basta et al. 2007) and has been proposed in Flanders (Khahzad and Reniers 2015) and the Walloon region of Belgium (Delvosalle et al. 2011, 2016)). The literature review has shown that the consequence-based approach is still the most common in EU countries. It has been applied in Finland, Luxemburg, Spain and Austria and just lately proposed in Greece (Christou et al. 1999; Sebos et al. 2010). It seems important to add that since the Toulouse accident in 2003, there has been a tendency of shifting from the consequence-based approach to the hybrid approach which combines the consequence-based and risk-based approaches. The hybrid approach has been lately adopted in France (Cahen 2006; Taveau 2010), Italy (Cozzani et al. 2006) and proposed in Romania (Török et al. 2019).

There is still much to develop and improve in the field of LUP in the vicinity of the Seveso establishments. The case study has revealed that it is worth considering the following improvements in relevant Polish legislation:

1. It is postulated that an extended description of the areas delineated for the location of the Seveso establishments should be introduced in the form of legal norms to be used in the spatial documents. This would require an extension of the set of standards regarding symbols of land-use, which is contained in Annex 2 to the ordinance (PJL 2003f). The extended description indicating the location of the Seveso establishments proposed in the Polish guidelines (Małaczyński et al. 2007, 2010) could be used here, i.e. “PS”, P standing for Industry (in Polish language “Przemysł”), S standing for Seveso.
2. It is recommended that an ordinance on the content of the SEA report for the plan should be introduced with a direct reference to the problem of major accident hazards, especially that a delegation to issue such an ordinance has been already included in the MEIA Act (2008a; 2018). In the period from 1995 to 2008, there were ordinances (PJL 1995, 2002b) regulating explicitly this issue, and the experience from that period could be used here.
3. It is suggested that a reference mathematical model for calculating the hazardous zone range should be introduced to the draft of the ordinance on safety distance determination (GLC 2019). The lack of a reference model may lead to big discrepancies in results of the hazard/risk assessment. Before making the decision about the reference model, there is a need either to perform quality evaluation of candidate models or to review results of the evaluation of the quality of such models described in the literature. A review of procedures for performing such evaluation can be found for example in Markiewicz (2013). In Poland, in the process of risk assessment of industrial accidents for the purposes of preparing the safety report required for UTE, usually three computer programmes are most often used: ALOHA, RIZEX-2, PHAST and EFFECTS (Wiśniewski et al. 2018). However, a set of potential candidates should not be limited to this software. There are many other computer tools available (Markiewicz 2012, 2018), including the EU Community ADAM module (Fabri and Wood 2019). Wiśniewski et al. (2018) have also reported the need to develop legal regulations concerning the assessment of major accident risk in Poland. They have recommended it in relation to LUP around hazardous establishments and in relation to analysis carried out for the purpose of preparing the safety reports.
4. It is postulated that changes to the SPLUM Act (PJL 2003a; PJL 2018a) should be introduced aimed at eliminating the location of Seveso establishments under the terms of decision on land development conditions and that an obligation be introduced to draw up the plan for the areas in which the Seveso establishments are located or siting of such plants is planned. This postulate is not new. It was risen for the first time in the unpublished manuscript within the EU River Shield project aiming to protect rivers against pollution caused by industrial accidents (Borysiewicz and Kacprzyk 2007). The decision on land development conditions has been introduced in Poland as a temporary measure that can be applied by the local authorities in situations when the plan is not available. Spatial planners consider that the excessive use of this instrument may obstruct rational urban development and functional zoning (Krajewska et al. 2014; Parysek 2016).

It is believed that the results of the study presented in this paper have useful implications for the control of major industrial accidents spatial policy-making and environmental management in Poland.
